# PIK3CA mutation testing and alpelisib use in metastatic breast cancer: a real-world data set

**DOI:** 10.2340/1651-226X.2026.45589

**Published:** 2026-05-11

**Authors:** Maja B. Drakenberg Danese, Christoffer P. Vannas, Henrik A. Fagman, Per O. Karlsson, Karolina F. Larsson

**Affiliations:** a Department of Oncology, Sahlgrenska University Hospital, Gothenburg, Sweden; b Department of Laboratory Medicine, Institute of Biomedicine, University of Gothenburg, Gothenburg, Sweden; c Department of Oncology, Institute of Clinical Sciences, Sahlgrenska Academy, University of Gothenburg, Gothenburg, Sweden; d Department of Clinical Pathology, Sahlgrenska University Hospital, Gothenburg, Sweden

**Keywords:** breast neoplasms, genetic testing, *PIK3CA*protein, precision medicine

## Introduction

Metastatic breast cancer (MBC) is an incurable disease, with a median overall survival (OS) of around three years [1]. Oestrogen receptor positive (ER+), human epidermal growth factor receptor 2 negative (HER2-) MBC accounts for approximately 70% of cases [2]. First line treatment with endocrine therapy (ET) with or without cyclin dependent kinase 4/6-inhibitors (CDK4/6i) has significantly improved median OS, which currently is approximately 5 years [3–5]. However, endocrine resistance, for example, by gene mutations in the PI3K/AKT/mTOR pathway or in the oestrogen receptor 1 (*ESR1*) gene, poses a significant clinical challenge and is associated with poorer prognosis [6–12].

The PI3K/AKT/mTOR pathway is involved in proliferation and reduced apoptosis in cancer cells. Activating mutations in the *PIK3CA* gene are present in 28–40% of ER+ HER2- MBC cases [13–19]. *PIK3CA* mutated MBCs show poorer chemotherapy response rates and OS [14, 20]. Consequently, targeting the PI3K pathway could suppress tumour progression and overcome endocrine resistance [21, 22].

The phase III SOLAR-1 trial and the prospective, single-arm BYLieve study, reported clinical benefit in patients with *PIK3CA* mutated ER+ HER2- MBC treated with fulvestrant and alpelisib. Both studies reported high frequencies of grade 3–4 adverse events (AEs) (65–76%), with hyperglycaemia and skin rashes being the most prevalent [13, 23].

The aim of this study was to describe the implementation of *PIK3CA* mutation testing, patients’ characteristics and the outcomes for patients treated with alpelisib in a publicly funded healthcare system in western Sweden.

## Material and methods

This descriptive retrospective study involved patients in western Sweden who were tested for PIK3CA mutations and, in some cases, subsequently treated with fulvestrant and alpelisib. The study was an observational study, aligned with EQUATOR guideline STROBE checklist (Supplemental material).

All material and methods can be found in supplementary file ‘Material and Methods’.

## Results

### Patient cohort and background characteristics

During the study period from October 2020 to February 2024, 346 patients were newly diagnosed with ER+ HER2- MBC in Region Vastra Gotaland. In addition, several previously diagnosed patients were under treatment. In total, 71 patients were tested for *PIK3CA* mutations during the study period, corresponding to approximately 20% of newly diagnosed patients. Of the tested patients, 58% had received adjuvant chemotherapy and 78% had received adjuvant ET (Table 1).

**Table 1:** Background characteristics of patients with ER+ HER2- metastatic breast cancer tested for PIK3CA mutation between October 2020 and February 2024 in Region Vastra Gotaland, western Sweden.

Patient characteristics	Total (n=71)	PIK3CA mutated (n=25)	PIK3CA wild type (n=46)	p-value
Adjuvant chemotherapy, n (%) Yes No Unknown	41 (58%) 25 (35% 5 (7%)	12 (48%) 11 (44%) 2 (8%)	29 (63%) 14 (30%) 3 (7%)	0.46
Adjuvant endocrine therapy, n (%) Yes Tamoxifen only Aromatase inhibitor only Both No Unknown*	55 (78%) 28 (39%) 19 (27%) 8 (11%) 10 (14%) 6 (9%)	20 (80%) 8 (40%) 9 (36%) 3 (12%) 3 (12%) 2 (8%)	35 (76%) 20 (44%) 10 (22%) 5 (11%) 7 (15%) 4 (9%)	0.71
Time from primary diagnosis to MBC, years Mean Median, (range)	6.6 5.1 (0–23.2)	5.8 4.7 (0–18.3)	7.1 5.2 (0–23.2)	0.39
Age group (MBC), n (%) <50 years 50-70 years >70 years	21 (30%) 43 (60%) 7 (10%)	4 (16%) 18 (72%) 3 (12%)	17 (37%) 25 (54%) 4 (9%)	0.13
Endocrine resistance**, n (%) Yes Primary Secondary No	37 (52%) 5 (7%) 32 (45%) 34 (48%)	15 (60%) 1 (4%) 14 (56%) 10 (40%)	22 (48%) 4 (9%) 18 (39%) 24 (52%)	0.33
Time from MBC diagnosis to PIK3CA mutation testing, years Mean Median (range)	3.2 2.7 (0.1–10.7)	3.0 2.6 (0.3–8.0)	3.3 2.8 (0.1–10.7)	0.54
Treatment lines in an advanced setting, prior to PIK3CA testing, n Median (range)	3 (1–14)	2 (1–6)	3 (1–14)	0.073
Chemotherapy in an advanced setting, prior to PIK3CA testing, n (%) Yes Nr of lines, median (range) No	46 (65%) 1 (0–8) 25 (35%)	15 (60%) 1 (1–3) 10 (40%)	31 (67%) 2 (1–8) 15 (33%)	0.039***
Endocrine therapy in an advanced setting, prior to PIK3CA testing, n (%) Yes Nr of lines, median (range) No	70 (99 %) 1 (0–3) 1 (1 %)	25 (100 %) 2 (1–3) 0 (0 %)	45 (98%) 1 (1–3) 1 (2 %)	0.46
CDK4/6 inhibitor treatment in an advanced setting, prior to PIK3CA testing, n (%) Yes No	67 (94%) 4 (6 %)	24 (96%) 1 (4 %)	43 (93%) 3 (7%)	0.66
Sites of metastasis at time of PIK3CA testing, n (%) Bone only Any visceral site Liver Lung/pleura Lymph node Brain Other sites	11 (15%) 60 (85 %) 37 (52%) 28 (39%) 19 (27%) 5 (7%) 16 (23%)	3 (12%) 22 (88%) 15 (60%) 9 (36%) 5 (20%) 0 (0%) 6 (24%)	8 (17%) 38 (83%) 22 (48%) 19 (41%) 14 (30%) 5 (11%) 10 (22%)	0.55 0.55 0.33 0.61 0.34 0.87 0.83

* For one patient some background information is missing because the primary breast cancer was treated abroad and not all medical records were available.

** Primary endocrine resistance defined as MBC diagnosis within 24 months after initiating curative endocrine therapy or progression within 6 months after initiating palliative endocrine therapy. Secondary endocrine resistance defined as MBC diagnosis during, but later than 24 months after initiating curative endocrine therapy or MBC within 12 months after finishing curative endocrine therapy or progression within 12 months after initiating palliative endocrine therapy.

*** The result did not reach statistical significance after Bonferroni correction, which set the adjusted threshold at p < 0.0083.

The median disease-free interval from diagnosis of primary breast cancer to MBC was 5.1 years (4.7 in *PIK3CA* mutated vs. 5.2 in wild type [WT]). There was a trend towards increased prevalence of *PIK3CA* mutations among older patients (84% > 50 years in mutated vs. 63% in WT, not significant) (Table 1).

Primary endocrine resistance was seen in five patients (7%) and secondary endocrine resistance in 32 patients (45%). Clinical endocrine resistance was more prevalent among patients with a *PIK3CA* mutation (60 vs. 48% in WT), however not significant. After MBC diagnosis, 99% of patients had received ET and 65% had received chemotherapy, with a median of 2.7 years and three treatment lines before being referred for *PIK3CA* mutation testing. A total of 94% of patients had received a CDK4/6i. No significant differences in previous treatments were observed between *PIK3CA* mutated and WT (Table 1).

### Outcomes of PIK3CA testing

*PIK3CA* mutations were detected in 25/71 patients (35%), aligning with the mutation frequency reported in the The Cancer Genome Atlas (32.6%) database [15, 16, 20]. Among the 25 patients with *PIK3CA* mutations, 75% had one of the mutations described in SOLAR-1. In 34% of cases, testing was performed on primary tumour tissue only and in 60% on metastatic tumour tissue only. Mutations were detected in 25 and 40% of cases, respectively, not significant. In 6% of cases, both primary tumour tissue and metastatic tumour tissue were tested, mutation statuses were concordant (Supplementary Table 1).

### Characteristics and clinical outcomes for patients treated with alpelisib

Eight of the 25 patients (32%) with a *PIK3CA* mutation received alpelisib in combination with fulvestrant. All patients treated with alpelisib had one of the mutations described in SOLAR-1. Of patients not treated with alpelisib, 12/17 (71%) had a SOLAR-1 mutation and 5/17 (29%) had other mutations (Supplementary Figure 1).

At MBC diagnosis, 6/8 (75%) patients treated with alpelisib were 50 years or older and 4/8 (50%) had endocrine resistance. Patients had received in median two treatment lines (range 1–3) in an advanced setting, including chemotherapy (5/8 (63%)) and ET (8/8 (100%)). At treatment start, 7/8 patients (88%) had a performance status of 0–2 and 5/8 (63%) were already on treatment with fulvestrant. Assessment of medical records for these patients revealed that treatment with fulvestrant was initiated before alpelisib was approved in Sweden and alpelisib was later added. Notably, the three patients with the longest response to alpelisib/fulvestrant had been treated with fulvestrant for the longest periods before adding alpelisib.

**Figure 1:**
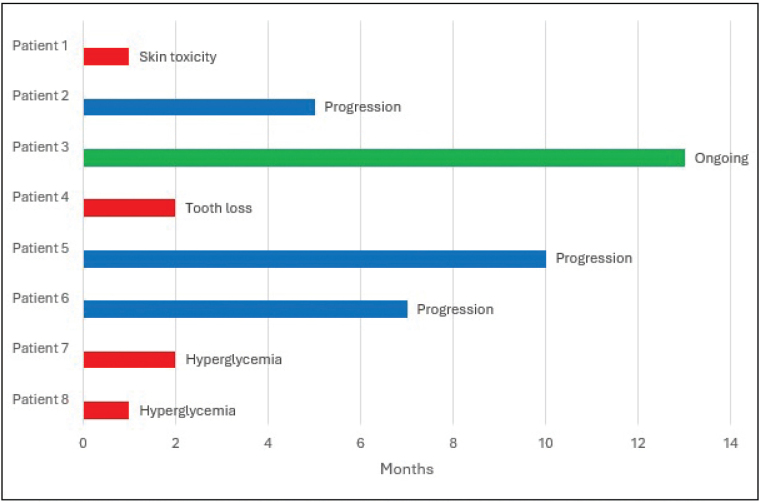
Swimmer’s plot of the clinical response to treatment with alpelisib and fulvestrant among eight patients with ER+HER2- metastatic breast cancer between October 2020 and February 2024 in Region Vastra Gotaland, western Sweden. Blue bars indicate patients stopping their treatment due to progression, red bars indicate patients stopping their treatment due to toxicity whereas green bars indicate patients still on treatment at data cut-off date. ER+HER2-: Oestrogen receptor positive human epidermal growth factor receptor 2 negative.

The most common reason for discontinuation was AEs (4/8 (50%)) followed by disease progression (3/8 (38%)). The median time on treatment was two months (range 1–10 months). Among patients who continued treatment until disease progression, the median time on treatment was seven months (range 5–10 months) (Figure 1).

## Discussion

In this study we report the clinical experience of *PIK3CA* mutation testing and treatment with alpelisib in western Sweden between October 2020 and February 2024, after alpelisib was approved in Sweden. The findings show that referral for testing and initiation of treatment has been limited. The challenges and moderate benefit seen in our cohort correlates with the downgrading of alpelisib in the latest ESMO Magnitude of Clinical benefit Scale (MCBS), due to toxicity and a lack of OS advantage [24].

The prevalence of *PIK3CA* mutations in our cohort (32%) is consistent with previous knowledge. However, in our cohort less than 20% of the patients with ER+ HER2- MBC became subject to *PIK3CA* testing. Since the SOLAR-1 trial did not reflect the standard of care, where treatment is typically initiated after progression on CDK4/6i, it may have been challenging for oncologists to find the optimal place for alpelisib in clinical practice, particularly when *PIK3CA* testing was not routinely performed.

Endocrine resistance was more prevalent, however not significant, among patients with a *PIK3CA* mutation (60 vs. 48%), which is concordant with the known role of PI3K in developing hormone-independent cancer cells [22].

Alpelisib was initiated at a far later stage in our setting compared to in SOLAR-1, likely explaining the lower response rates and tolerance to AEs. For patients who continued treatment until disease progression the median PFS was similar to results in SOLAR-1 and BYLieve trials [13, 23].

In SOLAR-1, *PIK3CA* mutation status was determined using a method that can detect eleven prevalent mutations activating the PI3K pathway [13]. Other sequencing methods, including the method used in this study, can detect additional mutations. Further research on the role of other *PIK3CA* mutations could potentially widen the indication for treatment with PI3K inhibitors. It remains to be determined whether this will increase the number of patients treated with alpelisib.

The PI3K inhibitor inavolisib and the AKT inhibitor capivasertib have both shown positive outcome results and been approved by EMA, with considerably lower prevalence of AEs than alpelisib [25, 26]. Additionally, the approval of trastuzumab deruxtecan for MBC patients with low and ultralow HER2 expression could possibly reduce the use of alpelisib in the future [27, 28].

### Limitations

This study is based on a small cohort and therefore it is impossible to draw significant conclusions but gives an indication on how alpelisib has performed in the clinical setting.

## Conclusion

We conclude that *PIK3CA* mutation testing was not successfully integrated into clinical routine in western Sweden, when alpelisib was introduced. To ensure timely and equitable access to new therapies, clear guidelines for mutation testing in routine care are needed.

For patients who tolerate treatment, responses were generally favourable, however many discontinued alpelisib due to toxicity. This underscores the need for improved strategies to prevent and manage AEs.

## Supplementary Material





## Data Availability

Pseudo‑anonymised data are not publicly available but may be presented upon reasonable request to the corresponding author.
