# Supplementary material for: PIK3CA mutation testing and alpelisib use in metastatic breast cancer: a real-world data set
Source: Acta Oncol. 2026 May 11;65:45589. doi: 10.2340/1651-226X.2026.45589 (PMC13170083; doi:10.2340/1651-226X.2026.45589)
Supplement: Supplementary file 1 [file AO-65-45589-s1.pdf]

Supplementary material has been published as submitted. It has not been copyedited, or typeset by Acta Oncologica

Material and Methods:

Patients

All patients with ER+ HER2- MBC in Region Vastra Gotaland, who underwent tumour genomic sequencing for ***PIK3CA*** mutations at the Department of Clinical Pathology at Sahlgrenska University Hospital between October 2020 and February 2024 were included. The total number of newly diagnosed patients in Region Vastra Gotaland with ER+ HER2- MBC during the same period was derived from the Swedish National Quality Registry for Breast Cancer.

Mutation testing

An amplicon-based next generation sequencing assay (ThermoFisher) covering all possible **DNA sequence variants** in 285 codons of the ***PIK3CA*** gene was used for ***PIK3CA*** mutation detection. Mutations assessed as "pathogenic" or "likely pathogenic" were reported.

Data collection

Clinical information such as treatments in the curative and advanced settings, disease-free interval between primary and metastatic disease and sites of metastasis was extracted from medical records. Prevalence of endocrine resistance was reported, where primary endocrine resistance was defined as MBC diagnosis within 24 months after initiating **adjuvant** endocrine therapy or progression within six months after initiating palliative endocrine therapy. Secondary endocrine resistance was defined as MBC diagnosis during, but later than 24 months after initiating **adjuvant** endocrine therapy or MBC within twelve months after finishing **adjuvant** endocrine therapy, or progression within twelve months after initiating endocrine therapy in the metastatic setting. ***PIK3CA*** mutation status and whether sequencing was performed on a primary tumour or a metastasis, including tissue type, was extracted from pathology reports. Among patients with a ***PIK3CA*** mutation, those who received alpelisib treatment were identified in the medical records.

Outcomes

Primary endpoint was time on treatment. Secondary endpoints were to describe the clinical characteristics of patients tested for ***PIK3CA*** mutations, assess the outcomes of the mutation testing and the reason for discontinuation of alpelisib treatment.

Statistical analyses

Descriptive clinical data was presented as case frequencies (n) with corresponding percentages of the total cohort. Continuous numerical descriptive characteristics were reported as mean and median values, including ranges. Comparisons of descriptive characteristics between patients with ***PIK3CA*** mutations vs wild type were performed using the Chi-Square test for categorical variables. For continuous variables, normality was assessed with the Shapiro-Wilk test. Normally distributed variables were analysed using independent two-sample t-tests, while non-normally distributed variables were analysed using Mann-Whitney U test. **To account for multiple comparisons ($m = 11$), a Bonferroni correction was applied by adjusting the threshold for statistical significance to $p < 0.0045$ (0.05/11). Accordingly, raw p-values are reported without adjustment.**
