# Supplementary material for: PIK3CA mutation testing and alpelisib use in metastatic breast cancer: a real-world data set
Source: Acta Oncol. 2026 May 11;65:45589. doi: 10.2340/1651-226X.2026.45589 (PMC13170083; doi:10.2340/1651-226X.2026.45589)
Supplement: Supplementary file 2 [file AO-65-45589-s2.pdf]

Supplementary material has been published as submitted. It has not been copyedited, or typeset by Acta Oncologica

Supplementary tables and figures

Tables

Supplementary table 1. Pathology results for patients with ER+ HER2- metastatic breast cancer tested for PIK3CA mutation between October 2020 and February 2024 in Region Vastra Gotaland, western Sweden.

Pathology data	Total (n=71)	PIK3CA mutated (n=25)	PIK3CA wild type (n=46)	p-value
Material tested for PIK3CA mutation, n (%)				0.4
Primary tumour (only)	24 (34%)	6 (24%)	18 (39%)	
Metastatis (only)	43 (60%)	17 (68%)	26 (57%)	
Both primary tumour and metastasis	4 (6%)	2 (8%)	2 (4%)	
Tumor location of material for PIK3CA mutation testing, n (%) Total	79*	29*	50*	
Breast	28 (35%)	9 (31%)	19 (38%)	0.35
Bone	8 (10%)	3 (10%)	5 (10%)	0.66
Liver	22 (28%)	11 (39%)	11 (22%)	0.08
Lung	6 (8%)	1 (3%)	5 (10%)	0.32
Lymph node	5 (6%)	0 (0%)	5 (10%)	0.087
Other location	10 (13%)	5 (17%)	5 (10%)	0.29

* For 8 patients PIK3CA mutation analysis was made on material from two sites, of which 4/8 (50%) had samples from primary tumours in the breast and from a metastatic lesion, 2/8 (25%) had samples from two different metastatic lesions, 1/8 (13%) had samples from a primary tumours in breast and a lymph node and 1/8 (13%) had two breast samples (uncertain if primary tumour and/or recurrence). The results were concordant in all these cases.

Figures

Supplementary figure 1. PIK3CA mutation variants detected among the 71 patients with ER+ HER2- metastatic breast cancer tested for PIK3CA mutations between October 2020 and February 2024 in Region Vastra Gotaland, western Sweden.

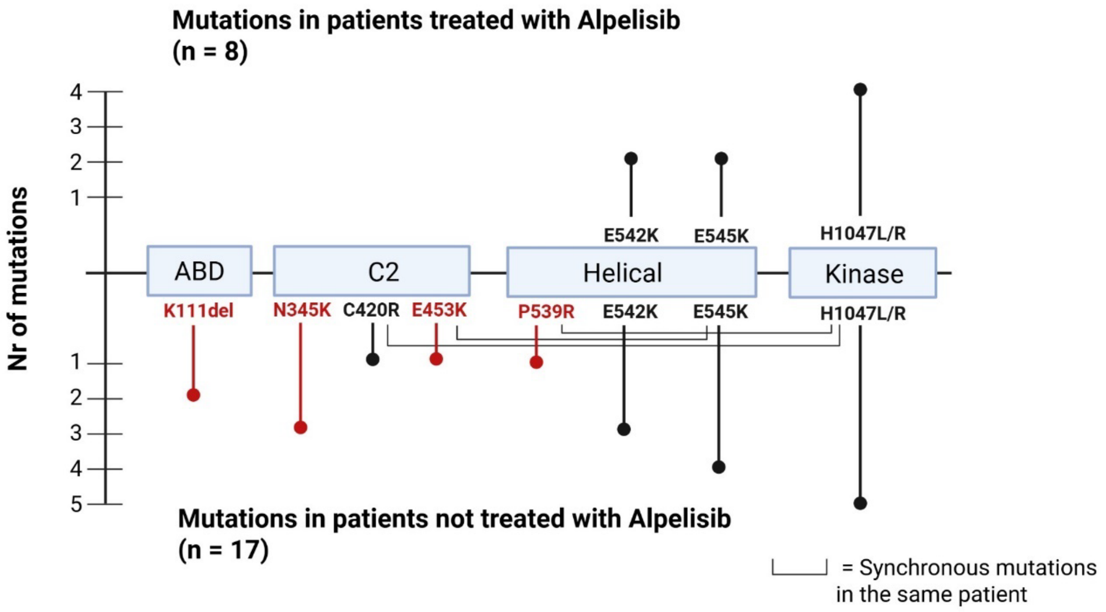

PIK3CA mutations detected in our cohort and location in the PIK3CA gene. The Adaptor-binding domain (ABD), C2-domain, Helical domain and Kinase functional domains of the PI3KCA gene have been highlighted. Upper panel indicate mutations in patients treated with Alpelisib (n = 8), whereas lower panel indicate mutations in patients not treated with Alpelisib (n = 17). Black text indicates mutation belonging to the SOLAR-1 panel whereas red text indicates mutations not belonging to the SOLAR-1 panel. Brackets indicate cases with multiple mutations occurring in the same patient.
